# Principles of adaptive element spacing in linear array antennas

**DOI:** 10.1038/s41598-021-84874-7

**Published:** 2021-03-10

**Authors:** Tanzeela Mitha, Maria Pour

**Affiliations:** Department of Electrical and Computer Engineering, The University of Alabama in Huntsville, Huntsville, AL 35899 USA

**Keywords:** Engineering, Electrical and electronic engineering

## Abstract

A novel approach to linear array antennas with adaptive inter-element spacing is presented for the first time. The main idea is based upon electronically displacing the phase center location of the antenna elements, which determine their relative coordinates in the array configuration. This is realized by employing dual-mode microstrip patch antennas as a constitutive element, whose phase center location can be displaced from its physical center by simultaneously exciting two modes. The direction and the amount of displacement is controlled by the amplitude and phase of the modes at the element level. This in turn facilitates reconfiguring the inter-element spacing at the array level. For instance, a uniformly-spaced array could be electronically transformed into a non-uniform one without any mechanical means. The proposed idea is demonstrated in two- and three-element linear antenna arrays. The technique has the potential to control the radiation characteristics such as sidelobe levels, position of the nulls, and the beamwidths in small arrays, which are useful for adaptively controlling the array performance in emerging wireless communication systems and radars.

## Introduction

Phased array antennas have attracted much attention in recent years because of their appealing capabilities to realize a variety of unique radiation characteristics such as high gain, low sidelobe levels, beam scanning, and null steering. The radiation characteristics mainly depend upon the element pattern, the excitation amplitude and phase, inter-element spacing, as well as the array geometry^1,2^. A number of well-defined techniques, namely the Taylor and Dolph–Chebyschev methods^1–4^, use tapered amplitude excitation on uniformly-spaced array antennas to reduce and control the sidelobe levels. Low sidelobe levels can also be achieved by optimizing the phase shifts of the uniformly-spaced array elements^5^. In 1961, Harrington^6^ proposed a novel technique of reducing the sidelobe levels of array antennas with uniform excitation by employing the method of non-uniform spacing of array elements. This technique was further investigated to design unequally-spaced array antennas with uniform^6–9^ as well as non-uniform excitation^10,11^ to improve the array performance, compared to uniformly spaced arrays^12–15^. It was demonstrated that the radiation characteristics, e.g., the position of the nulls, beamwidth, sidelobe levels, of the non-uniform arrays can be controlled by the location, magnitude and phase of their base elements. A vast variety of evolutionary algorithms such as genetic algorithm (GA), particle swarm optimization (PSO), and differential evolution (DE), were developed for the purpose of optimizing the element positions and excitation to reduce the computational cost^16–19^. This has led to the development of antenna arrays, realizing narrow beamwidths, null steering and reduced sidelobe levels by controlling the element position and excitation^20–23^. However, in order to achieve different desired radiation characteristics, it is required that the base element be physically displaced to a pre-determined position. As the element position varies per the requirement, the practical implementation becomes costly and complex. Therefore, a new research paradigm is needed to realize adaptive element spacing arrays over the course of the operation.

**Figure 1:**
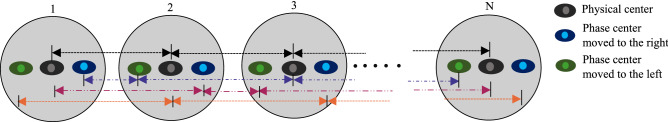
An N-element equally-spaced linear array with different phase center locations to generate different combinations of element spacing.

In this article, a novel approach is proposed for the first time to electronically change the array inter-element spacing by displacing the phase center locations of its antenna elements. It has already been established that the phase center location of a single, dual-mode circular microstrip patch antenna can be controlled by the amplitude and phase of mode content factors^24–28^. Such an intriguing and inspiring displaced phase center capability is utilized herein for the first time to realize adaptive element spacing in antenna arrays, whose physical elements are uniformly spaced. This article focuses on investigating the phase center displacement idea in two- and three-element linear arrays, consisting of the dual-mode circular microstrip patches. Nonetheless, the concept can be applied to N-element equally-spaced arrays as graphically illustrated in Fig. 1. The coordinates of each element are electronically changed by displacing the phase center location of the base element from its pre-determined central position to modify the element spacing in the array. First, a brief review of the phase center displacement in a single, dual-mode patch antenna is provided to understand the relation between the phase center location and element position and how it can be adaptively controlled. The dual-mode antenna is then considered as the base element for the two- and three-element linear array antennas where the phase center displacement technique is employed to adaptively change the element spacing without any physical displacement. Based on the analytical results of the three-element linear array, the array antenna along with its feeding network were simulated in a full-wave electromagnetic solver and fabricated using printed circuit board technology. The measured and simulated results are in good agreement with each other. They also verify the proposed idea in practice that the phase center displacement concept can be utilized to facilitate adaptive element spacing in equally-spaced array antennas to generate a variety of radiation patterns without any physical displacement.

### Phase center displacement of antenna element

**Figure 2:**
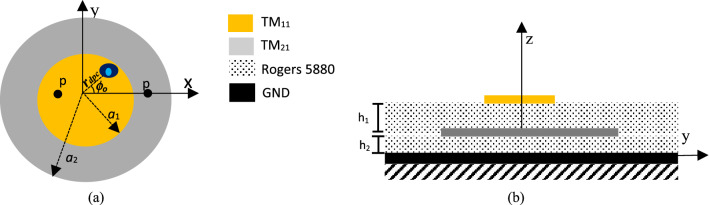
(**a**) Top- and (**b**) cross-section view of the stacked circular patch antenna operating at the TM_11_ and TM_21_ modes over an infinite ground plane. The patches have radii *a*_1_ = 5.4 mm and *a*_2_ = 9.2 mm, placed over Rogers 5880 dielectric with permittivity ε_*r*_ = 2.2; *h*_1_ = 1.6 mm and *h*_2_ = 1 mm and excited by SMA probes located at *p*_1_ = 2.7 mm and *p*_2_ = 5.8 mm; the “eye sign” represents the antenna phase center location, denoted by *r*_*dpc*_ and *ϕ*_o_ in the *xy-*plane.

The proposed concept of adaptive inter-element spacing in an array is realized by electronically displacing the phase center location of its base elements, which in turn alters the relative coordinates of the array elements. The phase center deviation is achieved by simultaneously exciting the first two azimuthal modes of circular microstrip antenna elements, detailed as follows. As per IEEE standard^29^, the phase center location of an antenna is the effective source of radiation, from which the spherical waves are formed in space with constant phase fronts over a sphere in the far-field region. Thus, any phase center displacement in an antenna element leads to an apparent change in its position. In general, the phase center location of a circular microstrip antenna is coincident with its physical center when it operates at a single mode, whether the dominant or a higher order mode^24^. However, if two or more modes are concomitantly excited, the phase center of the antenna may be displaced from the physical center of the patch. In order to demonstrate the phase center displacement, a stacked dual-mode circular patch antenna configuration is considered here. The antenna excites the TM_11_ and TM_21_ modes at the frequency of 10 GHz, using two probes located at *p*_1_ and *p*_2_ respectively along the x-axis, as shown in Fig. 2a. The patches and the ground plane are separated by thin layers of dielectric material (Rogers 5880) with a relative permittivity *ε*_*r*_ = 2.2 and heights *h*_1_ = 1.6 mm and *h*_2_ = 1 mm, respectively. The top- and side-views of this antenna are shown in Fig. 2.

**Figure 3:**
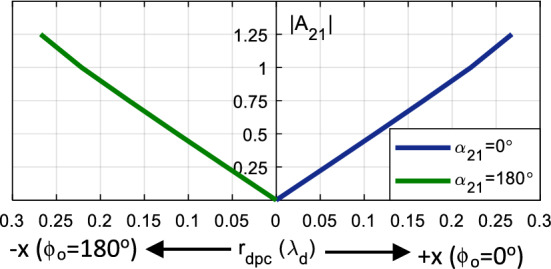
The effect of mode content factors on the phase center location for excitation phases of *α*_21_ = 0° and 180°.

**Figure 4:**
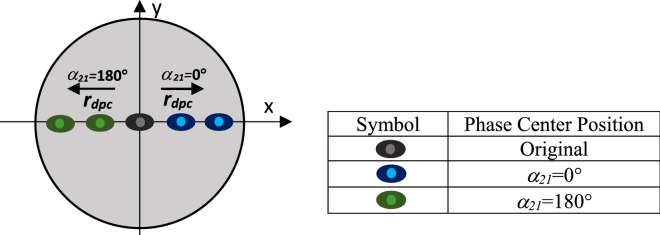
Pictorial representation of the phase center displacement in the base element. The phase center moves along the positive *x*-axis when *α*_21_ = 0°, i.e., TM_11_ + TM_21_ mode is excited, and along the negative *x*-axis when *α*_21_ = 180°, i.e. TM_11_–TM_21_ mode is excited.

To simplify the analysis, it is assumed that the patches are backed by an infinite ground plane. Based on the cavity model, the combined far-field equations of the radiation pattern for the *x*-polarized TM_11_ and TM_21_ modes are given by:

1 $${E}_{\theta }=-j\frac{{e}^{-j{k}_{0}r}}{r}{e}^{j{k}_{0}{r}_{dpc}sin\theta \mathrm{cos}\left(\phi -{\phi }_{0}\right)}{e}^{j{k}_{0}{z}_{0}cos\theta } \left\{\left[{J}_{0}\left({u}_{1}\right)-{J}_{2}\left({u}_{1}\right)\right] cos\phi +j{A}_{21}\left[{J}_{1}\left({u}_{2}\right)-{J}_{3}\left({u}_{2}\right)\right]cos2\phi \right\}$$

2 $${E}_{\phi }=-j\frac{{e}^{-j{k}_{0}r}}{r}{e}^{j{k}_{0}{r}_{dpc}sin\theta \mathrm{cos}\left(\phi -{\phi }_{0}\right)}{e}^{j{k}_{0}{z}_{0}cos\theta } \left\{\left[{J}_{0}\left({u}_{1}\right)+{J}_{2}\left({u}_{1}\right)\right] sin\phi +j{A}_{21}\left[{J}_{1}\left({u}_{2}\right)-{J}_{3}\left({u}_{2}\right)\right]sin2\phi \right\}cos\theta $$

where the second and third exponential terms represent the antenna phase center location in polar coordinates, denoted by (*r*_*dpc*_, *ϕ*_*o*_, *z*_*o*_); *J* is the Bessel function of the first kind with associated eigenvalues of 1.8412 and 3.0542 for the TM_11_ and TM_21_ modes, respectively; *A*_21_ is the normalized excitation ratio (TM_21_ to TM_11_ mode), which is also known as the mode content factor. It is a complex number in general, defined as *A*_21_ = *|A*_21_*|*∠*α*_21_, where *|A*_21_*|* and *α*_21_ represent the magnitude and the phase shift between the two modes, respectively. The arguments of the Bessel functions, i.e.,* u*_1_ and *u*_2_, are defined by:

3 $$ \begin{aligned} u_{1} & = k_{0} a_{1} sin \theta \\ u_{2} & = k_{0} a_{2} sin \theta \\ \end{aligned} $$

here *k*_0_ is the wave number and *a*_1_ and *a*_2_ are the physical radii of the TM_11_ and TM_21_ patches with values 5.4 mm and 9.2 mm, respectively, at the designed frequency of 10 GHz. The radii (*a*_*n*_) of a TM_n1_ mode circular patch can be calculated using^1^:

4 $${a}_{n}=\frac{{\mathcal{X}}_{n1}^{{\prime}}{\lambda }_{0}}{2\pi \surd {\varepsilon }_{r}}$$

where λ_0_ is the free space wavelength at the resonant frequency and *χ*′_n1_ is the zero of the derivative of the Bessel function of first kind and order *n*^th^. In the *ϕ* = 0° plane, both TM_11_ and TM_21_ modes contribute to the *E*_*θ*_ radiation pattern. Thus, the *E*_*θ*_ radiation pattern is further investigated. When only the TM_11_ mode is excited, the antenna emits a broadside radiation pattern with a uniform phase distribution, indicating that the phase center is located at the physical origin. By adding the TM_21_ mode to the dominant TM_11_ mode, the far-field phase distribution over the main beam is no longer uniform, implying that the phase center has been displaced, whose coordinates are represented by (*r*_*dpc*_, *ϕ*_*o*,_ *z*_*o*_) in (1) and (2). As the thickness of the substrate is very small with respect to the wavelength, the displacement along the *z* direction is considered negligible, i.e. *z*_0_ ~ 0. Therefore, for a given *A*_21_, *r*_*dpc*_ and *ϕ*_*o*_ can be uniquely determined such that they make the far-field phase distribution uniform. As a result, the direction and the amount of displacement of the phase center depend on the magnitude and phase of the mode content factor *A*_21_. For all non-zero amplitude excitations and non-quadrature phase differences of ± 90°, the phase center is displaced along the *x*-axis. To obtain an equal amount of displacement, but in the opposite direction, one may only change the polarity of the applied phase shift. For a given |*A*_21_|, the maximum phase center displacement is realized when the phase shift between the modes is 0° and 180°, which moves the phase center away from the center along the + *x* and − *x* axis, i.e.,* ϕ*_o_ = 0° and *ϕ*_o_ = 180°, respectively^24^. For further clarification, the phase center location of the dual-mode antenna is plotted in Fig. 3, for different |*A*_21_| with in- and out-of-phase excitation of *α*_21_ = 0° and *α*_21_ = 180°, respectively. As the mode content factor increases, the phase center moves farther away from the origin. This is also graphically illustrated in Fig. 4, wherein the potential phase center locations are denoted by “eye signs” and they are color-coded throughout this article. That is, the black eye sign represents the physical center of the antenna, implying that only the TM_11_ mode is excited, and the blue and green eyes accord with the phase center locations due to the dual-mode excitation, which are displaced along the + *x* and − *x* axis, respectively.

The phase center displacement concept detailed above will be applied to two- and three-element arrays, respectively, to demonstrate the underlying principles of adaptive element spacing in linear arrays.

### Two-element array

**Figure 5:**
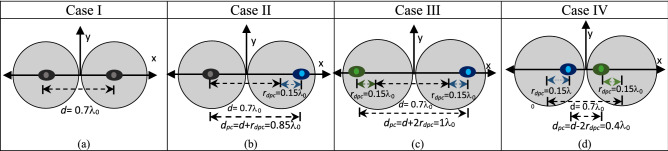
Structure and position of phase center of the two-element array with physical element spacing of *d*  = 0.7λ_0_ for (**a**) Case I: both elements excite only the TM_11_ modes with |*A*_21_| = 0∠0°, (**b**) Case II: *A*_21_ = 1∠0° for the right element and *A*_21_ = 0∠0° for the left element, (**c**) Case III: *A*_21_ = 1∠0° for the right element and *A*_21_ = 1∠180° for the left element, and (**d**) Case IV: *A*_21_ = 1∠180° for the right element and *A*_21_ = 1∠0° for the left element.

**Figure 6:**
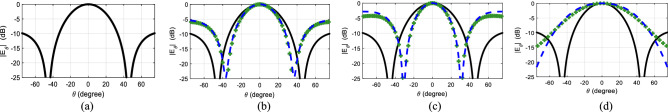
Comparison of the radiation patterns of the dual-mode (TM_11_ and TM_21_) two-element array antenna configurations of (**a**) Case I, (**b**) Case II, (**c**) Case III, and (**d**) Case IV, as shown in Fig. 5; solid black lines: TM_11_ mode, dashed blue lines: dual-mode, green plus symbols: physically displaced.

In this section, a two-element linear array is studied, whose base elements are the dual-mode circular patch antennas discussed above. These elements are placed such that their physical centers are 0.7λ_0_ apart, where λ_0_ is the free space wavelength at 10 GHz. The phase center displacement for the two elements is investigated for the following four cases, as illustrated in Fig. 5. In Case I, only the TM_11_ mode of both antenna elements are excited, i.e. *A*_21_ = 0∠0°. As no phase center displacement takes place due to the single-mode excitation, the phase centers, represented by the black eyes, are located at the physical centers of the antennas. The radiation pattern of this arrangement is shown in Fig. 6a. The pattern has a half-power beamwidth (HPBW) of 39° and the first nulls are located at ± 45.5°. This case serves as the reference case and the radiation patterns of the following three cases are compared to it to highlight the potential changes that occur due to the phase center displacement and thus to the element spacing within the array.

Case II represents the scenario where the phase center location of the right element is electronically displaced by *r*_*dpc*_ = 0.15λ_0_ along the + *x* axis by exciting the TM_21_ mode in-phase with the dominant mode with *A*_21_ = 1∠0°. The left antenna only excites the dominant TM_11_ mode and its phase center location thus remains unchanged at the physical patch center. This is illustrated in Fig. 5b. Therefore, the effective element spacing in Case II now becomes greater than its physical one of *d* = 0.7λ_0_ and is equal to *d*_*pc*_ = *d* + *r*_*dpc*_ = 0.7λ_0_ + 0.15λ_0_ = 0.85λ_0_. The corresponding radiation pattern of Case II is plotted in Fig. 6b and compared with that of the reference case. The larger element spacing occurred in Case II due to the displaced phase center of its right element increases the overall length of the array. This in turn changes the location of the null to ± 36.8°, the HPBW to 33.4° and increases the sidelobe level of the array radiation pattern. This pattern closely resembles that of a single-mode, two-element array antenna with a physical element spacing of 0.85λ_0_, which is also plotted in Fig. 6b for comparison, marked by green ‘+’ symbols.

In Case III, the phase centers of both elements are displaced away from each other. That is, the phase center of the left element is further pushed to the left by *r*_*dpc*_ = 0.15λ_0_, by exciting the TM_21_ mode 180° out of phase with the TM_11_ mode with *A*_21_ = 1∠180°, whereas the phase center of the right element is shifted to the right by *r*_*dpc*_ = 0.15λ_0_ by in-phase exciting the two modes with *A*_21_ = 1∠0°. Thus, the distance between the elements now increases to *d*_*pc*_ = *d* + 2*r*_*dpc*_ = 1λ_0_, as illustrated in Fig. 5c. The radiation pattern of this arrangement is plotted in Fig. 6c. This pattern is similar to that of a single-mode, two-element array antenna with a physical element spacing of 1λ_0_, as shown in Fig. 6c. As observed, the beamwidth becomes narrower as the HPBW shrinks to ~ 29° and the null locations change to ± 30.8°, which is attributed to the larger effective length of the array compared to the reference case.

In Case IV, the phase center locations of both elements are electronically pushed inwards by *r*_*dpc*_ = 0.15λ_0_ on each side, thus reducing the distance between the elements from *d* = 0.7λ_0_ to *d*_*pc*_ = *d* − 2*r*_*dpc*_ = 0.4λ_0_, as depicted in Fig. 5d. This is accomplished by exciting the higher order mode in-phase with the dominant mode in the left element (TM_11_ + TM_21_) and out-of-phase in the right element TM_11_–TM_21_. That is, the mode content factors of the left and right elements are *A*_21_ = 1∠0° and *A*_21_ = 1∠180°, respectively. The radiation pattern of this configuration is shown in Fig. 6d, where a wider beam with HPBW of 67.6° is now realized due to the smaller effective length of the array. The similarity between this pattern and that of a two-element array antenna with a physical element spacing of 0.4λ_0_ is highlighted by their respective curves overlaid in Fig. 6d. Thus, the phase center displacement technique can be used to electronically alter the element spacing and thus the overall length of this two-element antenna array.

From the above cases, it can be established that the distance between the phase center locations of the base elements in an array can be varied by exciting the higher order mode in- and out-of-phase with the dominant mode. This is achieved by varying the magnitude and phase of the mode content factor, i.e. |*A*_21_| and *α*_21_. The radiation characteristics for Cases I–IV are summarized in Table 1. The gain reduction in Cases II–IV is attributed to the reduction in the element gain due to the excitation of the higher order TM_21_ mode along with the fundamental TM_11_ mode.

**Table 1:** Radiation characteristics of the two-element linear array antennas.

	Case I	Case II	Case III	Case IV
HPBW	39°	33.4°	29°	67.6°
Null location	± 45.5°	± 36.8°	± 30.8°	± 90°
Gain	8.29 dBi	7.52 dBi	6.67 dBi	6.44 dBi

We can thus conclude that the displaced phase center technique can be used to electronically change the element spacing without any physical movement and be eventually used to realize a non-uniform array in larger array configurations. This idea of changing the apparent location of the base element by displacing its phase center can be used in N-element arrays to create adaptive element positioning without any mechanical means. It would help bypass the physical constraints of element rearrangement that is necessary to achieve the desired radiation pattern. In order to demonstrate this further, the proposed concept is applied to a three-element array where its effect on the null position, sidelobe level, and beamwidth is investigated.

### Three-element array

In this section, the proposed concept is further examined in a three-element array antenna. The base elements of the array are placed such that their physical centers are *d* = 0.7λ_0_ apart. In order to study the effect of displacing the phase center of the array elements on the element position, the overall length of the array, and the resulting radiation patterns, four cases (V to VIII) are considered as follows.

**Figure 7:**
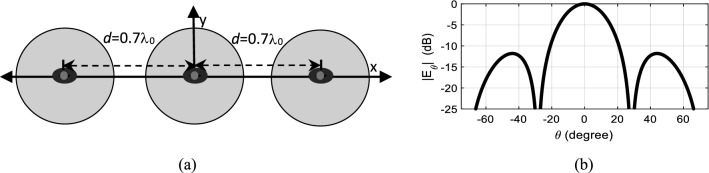
Case V (**a**) structure and (**b**) radiation pattern of dual-mode, three-element array antenna physically placed *d* = 0.7λ_0_ apart, with all three elements exciting TM_11_ mode, i.e., *A*_21_ = 0∠0°.

In Case V, the phase center locations of all the elements are at their physical centers as only the dominant mode is excited in each element, i.e.,* A*_21_ = 0∠0°. The array configuration along with its radiation pattern is shown in Fig. 7. The main beam has a HPBW of 24.8° and the first nulls are located at ± 28.3°. This case is considered as the reference case, to which the remaining three cases are compared.

**Figure 8:**
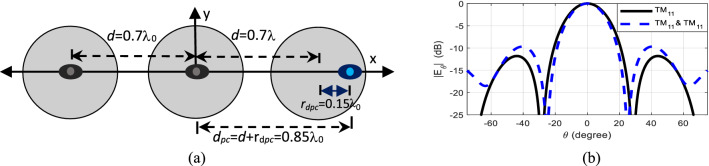
Case VI (**a**) structure and (**b**) radiation pattern of dual-mode, three-element array antenna physically placed *d* = 0.7λ_0_ apart, with phase center of the right element shifted to the right by *r*_*dpc*_ = 0.15λ_0_ when *A*_21_ = 1∠0°.

In Case VI, the phase center of the right element is shifted to the right by *r*_*dpc*_ = 0.15λ_0_ by setting its mode content factor to *A*_21_ = 1∠0°. The phase center locations of the remaining two elements are kept at their physical centers by only exciting their dominant TM_11_ mode, i.e. *A*_21_ = 0∠0°. As the phase center of the right element is displaced, its relative position in the array is shifted to the right by *r*_*dpc*_, thus now forming an unequally spaced array. As a result, the overall length of the array increases causing a change in the overall radiation pattern. This is illustrated by the variation in the sidelobe level from − 11.8 to − 9 dB, as well as changes in the position of the nulls from ± 28.3° to ± 25.6° and HPBW from 24.8° to 22° as shown in Fig. 8. This pattern closely resembles that of a three-element non-uniform array, with element spacing 0.7λ_0_ on the left side and 0.85λ_0_ on the right, which is omitted here for brevity.

**Figure 9:**
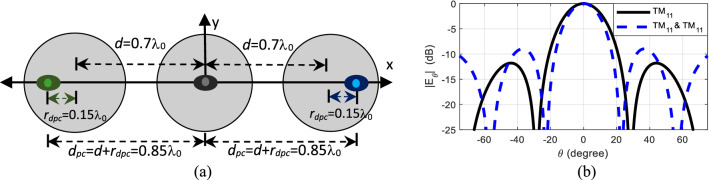
Case VII (**a**) structure and (**b**) radiation pattern of dual-mode, three-element array antenna physically placed *d* = 0.7λ_0_ apart, with phase center of right element and left elements displaced away from the center of the axis by *r*_*dpc*_ = 0.15λ_0_, with mode content factors *A*_21_ = 1∠0° and *A*_21_ = 1∠180° respectively.

In Case VII, both the right and left elements’ phase center locations are displaced away from the axis origin by *r*_*dpc*_ = 0.15λ_0_, by exciting the higher order mode in-phase and out-of-phase with the dominant mode, i.e. *A*_21_ = 1∠0° and *A*_21_ = 1∠180°, respectively. This creates a uniformly-spaced array with element spacing of 0.85λ_0_ as depicted in Fig. 9a. Thus, the overall length of the array increases without any physical displacement and its effect on the radiation pattern is shown in Fig. 9b. It is observed that the null location shifts from ± 28.3° to ± 22.7°, sidelobe level increases from − 11.8 to − 8.9 dB, and HPBW reduces from 24.8° to 20.8°, similar to the previous case. Nonetheless, this case study may serve as the basis for larger array implementations with adaptive element spacing.

**Figure 10:**
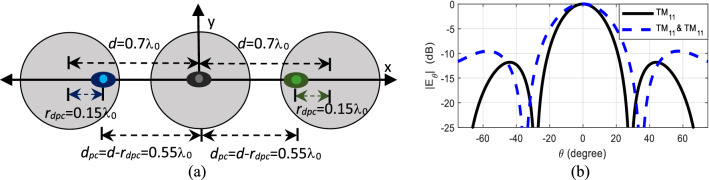
Case VIII (**a**) structure and (**b**) radiation pattern of dual-mode, three-element array antenna physically placed *d* = 0.7λ_0_ apart, with phase center of right element and left elements displaced towards the center of the axis by *r*_*dpc*_ = 0.15λ_0_, with mode content factors *A*_21_ = 1∠180° and *A*_21_ = 1∠0° respectively.

In Case VIII, the phase center locations of the right and left elements are moved inwards by *r*_*dpc*_ = 0.15λ_0_ on each side. This is achieved by exciting the higher order mode out of phase with the dominant mode TM_11_–TM_21_ in the right element, i.e. *A*_21_ = 1∠180°, and in-phase TM_11_ + TM_21_ in the left element, i.e. *A*_21_ = 1∠0°. This makes a uniformly-spaced array with element spacing 0.55λ_0_, thus decreasing the overall length of the array and leading to a change in the radiation pattern, as shown in Fig. 10. The null location shifts from ± 28.3° to ± 34.7°, sidelobe level increases from − 11.8 to − 9.5 dB, and HPBW increases from 24.8° to 32°, as now the effective array length becomes virtually smaller.

It is observed from the above cases that different periodic and aperiodic configurations can be realized using the displaced phase center technique without physically rearranging base elements. Each configuration led to a change in the overall radiation characteristics. The radiation characteristics of the arrays for Cases V–VII are summarized in Table 2. The proposed displaced phase center technique can be utilized in larger array antennas to develop different periodic and aperiodic configurations that alter the radiation patterns and achieve unique characteristics such as low sidelobe levels to suppress interference signals, null steering for anti-jamming applications, high gain and beam scanning for tracking targets in radars.

**Table 2:** Radiation characteristics of the three-element linear array antennas.

	Case V	Case VI	Case VII	CaseVIII
HPBW	24.8°	22°	20.8°	32°
Null location	± 28.3°	± 25.6°	± 22.7°	± 34.7°
Gain	10.25 dBi	9.62 dBi	9.19 dBi	9.06 dBi

It should be noted that the aforementioned dual-mode elements generate a broadside radiation pattern despite any phase center displacement, as the element pattern remains stationary in space. The phase center displacement is carried out within the element, which then changes its relative coordinate in the array. This in turn facilitates the realization of different aperiodic arrays to control the beamwidth in small arrays, sidelobe levels and null locations. Thus, the proposed phase center displacement technique does not hinder the beam scanning capability of the array in any way, as the main beam can be steered in a similar manner as in conventional phased array antennas by applying proper phase shifts at the array level.

## Methods

**Figure 11:**
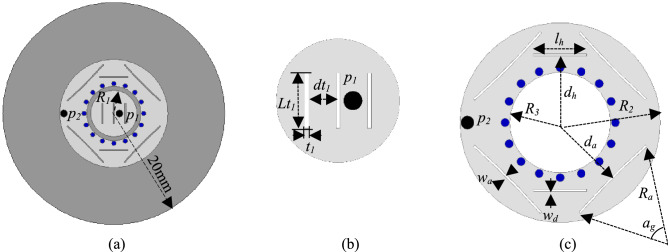
Geometry of the single-layer, dual-mode circular microstrip patch antenna operating at the TM_11_ and TM_21_ modes, where *R*_1_ is the radius of the TM_11_ patch, and *R*_2_ and *R*_3_ are the outer and inner radii of TM_21_ ring patch, respectively; *p*_1_ and *p*_2_ represent probe locations. (**a**) Top view, (**b**) TM_11_ circular patch, (**c**) TM_21_ ring patch.

The stacked configuration of the dual-mode circular patch antenna, assumed in the preceding analytical investigations in the three-element array design, increases the complexity of its fabrication and assembly in practice. Thus, to alleviate the manufacturing process of an adaptive three-element linear array, a single-layer dual-mode patch antenna^30^ with the displaced phase center property is used as the base element of the array. The single-layer antenna element is printed on a 1.52 mm-thick Rogers RO3003 substrate with the dielectric constant *ε*_*r*_ = 3. The base elements of the array are composed of a central circular patch with radius *R*_1_ = 4.16 mm, exciting the TM_11_ mode and a concentric short-circuited ring patch with outer radius *R*_2_ = 9.751 mm and inner radius *R*_3_ = 4.9 mm exciting the TM_21_ mode with probe *p*_1_ and *p*_2_, respectively, at a frequency of 10 GHz. The central circular patch of the base element has three vertical slits of thickness *t*_1_ = 0.25 mm and length *Lt*_1_ = 3.7 mm that are apart by a distance *dt*_1_ = 2.1 mm to make the design compact. A detailed illustration of the base element is provided in Fig. 11. The inner edge of the ring patch is short-circuited to the ground through the substrate with 16 metallic vias with diameter *v*_*d*_ = 0.81 mm to improve the isolation between the TM_11_ and TM_21_ modes. Furthermore, four symmetric arc slits of width *w*_*a*_ = 0.2 mm and angle *a*_*g*_ = 7.6° are etched on the ring patch to facilitate the TM_21_ mode purity. The arc slits are at a distance *d*_*a*_ = 7.3 mm from the central circular patch and are curved as per an outer circle of radius *R*_*a*_ = 55 mm. Additionally, the short-circuited concentric ring patch has two horizontal slits with length *l*_*h*_ = 5.2 mm and width *w*_*d*_ = 0.2 mm, located at distance *d*_*h*_ = 6.5 from the center, to improve the purity of the TM_11_ mode and suppress its orthogonal mode.

**Figure 12:**
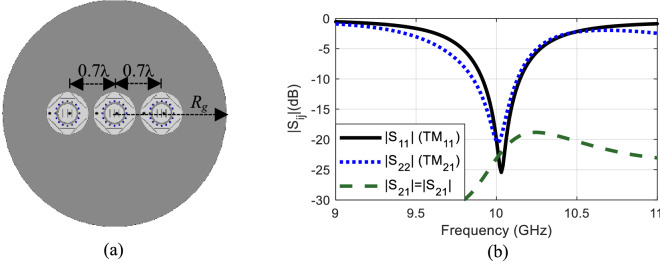
(**a**) Geometry of the single-layer, three-element array antenna operating at the TM_11_ and TM_21_ modes, over a finite ground plane of R_g_ = 60 mm; the patches are etched on 1.52 mm-thick Rogers RO3003 dielectric with permittivity ε_*r*_ = 3. (**b**) Simulated scattering parameters of the dual-mode base elements exciting the TM_11_ and TM_21_ modes.

Based on this single-layer element design, a three-element equally-spaced linear array antenna with the phase center displacement property was designed using the finite-element based full-wave solver ANSYS HFSS^31^. The antenna array is printed on a circular, 1.52 mm-thick Rogers RO3003 substrate with dielectric constant *ε*_*r*_ = 3 and radius *R*_*g*_ = 60 mm. To reduce the mutual coupling between the adjacent ports the base elements of the array are placed 0.7λ_0_ apart. The geometry of this single-layer, three-element design is illustrated in Fig. 12. Each element of the array is connected to two probes that excite the TM_11_ and TM_21_ modes at the frequency of 10 GHz. The simulated scattering parameters of the dual-mode element within three-element linear array are shown in Fig. 12b, where |S_12_| or |S_21_| represents the mutual coupling between the TM_11_ and TM_21_ modes in the base element and |S_11_| and |S_22_| are the reflection coefficients of the ports exciting the TM_11_ and TM_21_ modes, respectively. The mutual coupling between the modes are well below − 20 dB at 10 GHz thus contributing to small interference between them. The dual-mode circular patches have a narrow impedance bandwidth of 2.2% and 2.8% for the TM_11_ and TM_21_ modes, respectively.

In order to verify the proposed idea, the full-wave simulation and measurement analysis of Cases V and VIII in the three-element linear array antenna are carried out with the help of the feeding network designed below.

### Feeding network

**Figure 13:**
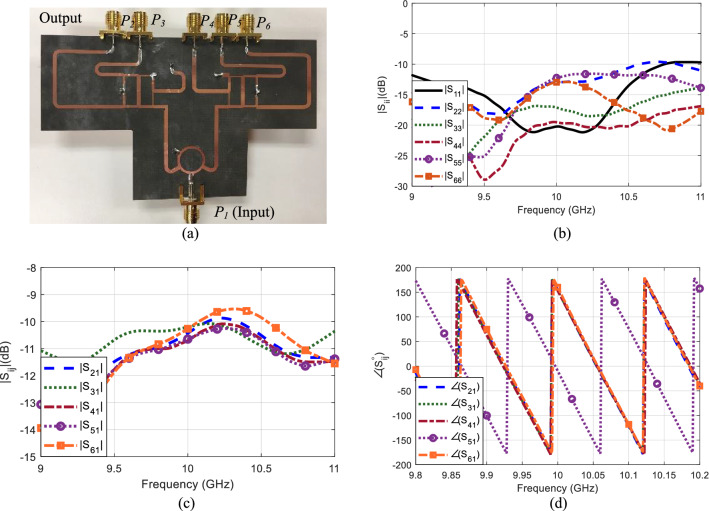
(**a**) A photograph of the fabricated feeding network with one input port *P*_1_ and five output ports *P*_2–6_. Measured (**b**) reflection coefficients, (**c**) insertion loss and (**d**) phase difference between the ports.

The phase center location of the base elements in the three-element array are modified using the 1:5 power splitter represented in Fig. 13a. This power splitter is composed of one 1:2 in-phase coupler^32^ and two 1:3 power splitters^33^. The power divider is etched on 0.508 mm-thick Rogers 5880 substrate with dielectric constant *ε*_*r*_ = 2.2. The feeding network is designed to operate at a central frequency of 10 GHz. The input and output ports are connected to 50 Ω transmission lines of thickness 1.565 mm. This feeding network has one input port (*P*_1_), 5 output ports (*P*_2–6_), 5 isolation ports and one matched port. The isolation ports and matched port are connected to 50 Ω loads. The feeding network is designed such that the radiating elements connected to the output ports 2, 3, 4 and 6 are excited by signals of equal magnitude and 0° phase shift at the operating frequency. Port 5, on the other hand, provides a signal with equal magnitude but 180° phase shift at the same frequency. The feeding network has an insertion loss of ~ − 10.5 dB at 10 GHz. The measured reflection coefficients, insertion loss and phase shifts of all the input and output ports are summarized in Fig. 13b,c,d.

### Measured results

**Figure 14:**
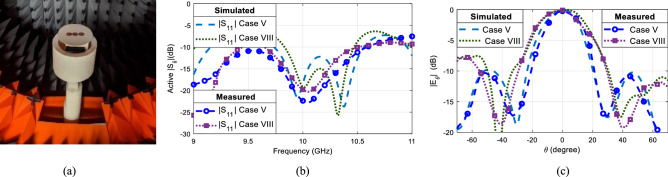
(**a**) Three-element single-layer array under test in an anechoic chamber and its measured and simulated (**b**) active scattering parameters, (**c**) radiation patters for Case V and Case VIII.

Based on the full-wave simulation of the design, a three-element single-layer array antenna was fabricated using the printed circuit board (PCB) technology. Each element of the array was connected to two SMA probes to excite the TM_11_ and TM_21_ modes. For Case V, only the TM_11_ modes of each element needed to be excited. Hence, the TM_11_ ports of each element of the array were connected to ports *P*_3_, *P*_4_ and *P*_6_ of the feeding network, which supplied signals with equal magnitude and 0° phase shift. The remaining output ports of the feeding network as well as the TM_21_ ports of the antenna array were connected to matched loads. This entire setup with the antenna array and feeding network was first simulated in HFSS and then the fabricated model was assembled and measured in the spherical near-field anechoic chamber at The University of Alabama in Huntsville. Figure 14a shows the assembled antenna array prototype under test in the anechoic chamber. For Case VIII, the TM_21_ modes of the right and left element of the array were excited along with the TM_11_ modes of all the three-elements. The left element TM_21_ mode was excited with a signal of equal magnitude and 0° phase shift by connecting it to port *P*_2_ of the feeding network, whereas the TM_21_ mode in the right element of the array was excited with a signal of equal magnitude and 180° phase shift by connecting it to port *P*_5_ of the feeding network. Consequently, the phase centers of the edge elements moved inwards, decreasing the overall electric length of the array. This altered the overall radiation pattern of the array antenna. The simulated and measured active scattering parameters of the array with the feeding network for both Cases V and VIII are shown in Fig. 14b. The measured and simulated active scattering parameters are below − 18 dB at 10 GHz. The difference in the simulated and measured active scattering parameters are due to the grounding mismatch in the simulated model and the manual assembly of the fabricated antenna with the feeding network. The measured radiation patterns of the assembled array antenna are compared to the simulated ones in Fig. 14c at the frequency of 10 GHz for Cases V and VIII. Overall, the radiation pattern results of the fabricated antenna are in good agreement with the simulated ones. In particular, it is clearly observed how the null locations and the beamwidths are impacted by adaptively changing the element spacing in the three-element array. The slight variations observed in the measured and simulated radiation patterns are mainly attributed to the inaccuracies in the antenna assembly and the supporting mast used for the measurement.

## Conclusion

The concept of adaptive element spacing in linear array antennas was investigated in this article for the first time, which was inspired by the intriguing capability of a dual-mode circular patch antenna to displace the phase center location away from its physical center, while maintaining its broadside radiation pattern. This paved the way to electronically change the array element spacing through associating the relative position of the antenna element in an array configuration to its respective phase center location. As a result, the transformation of a uniformly-spaced array into a non-uniform one was made possible with the proposed technique without any mechanical means. As a proof of concept, thorough investigations were carried out in two- and three-element uniform arrays, consisting of the dual-mode elements with a fixed physical element spacing of 0.7λ_0_. The results demonstrated that different element spacing ranging from 0.55λ_0_ to 0.85λ_0_ could be realized by controlling the magnitude and phase of the dual-mode elements without mechanically moving the elements. The distance between the elements was varied electronically in different case studies to create periodic and aperiodic array antennas that could control the radiation characteristics. Thus, the proposed technique can be used in larger arrays to adaptively control their radiation characteristics, such as sidelobes, grating lobes, and nulls, without facing the physical constraints on rearranging the antenna elements to pre-determined positions for a given requirement. The proposed reconfigurable element-spacing array antenna has the potential to transform the next generation phased array antennas in radar and remote sensing applications.
